# Quality of Life and Intention to Return among Former Residents of Tomioka Town, Fukushima Prefecture 9 Years after the Fukushima Daiichi Nuclear Accident

**DOI:** 10.3390/ijerph17186625

**Published:** 2020-09-11

**Authors:** Makiko Orita, Yasuyuki Taira, Hitomi Matsunaga, Masaharu Maeda, Noboru Takamura

**Affiliations:** 1 Department of Global Health, Medicine and Welfare, Atomic Bomb Disease Institute, Nagasaki University, Nagasaki 8528523, Japan; y-taira@nagasaki-u.ac.jp (Y.T.); hmatsu@nagasaki-u.ac.jp (H.M.); takamura@nagasaki-u.ac.jp (N.T.); 2 Department of Disaster Psychiatry, School of Medicine, Fukushima Medical University, Fukushima 960-1247, Japan; masagen@fmu.ac.jp

**Keywords:** Fukushima Daiichi nuclear accident, intention to return, quality of life, recovery efforts from the nuclear accident

## Abstract

We evaluated the association between health-related quality of life (HR-QOL) and intent to return home among former residents of Tomioka Town, Fukushima Prefecture 9 years after the Fukushima Daiichi nuclear accident to support the recovery of the community after the accident. We conducted a questionnaire survey asking residents about their intention to return to their original home, risk perception for radiation exposure, HR-QOL using the HR-QOL Short Form 8 (SF-8), and sense of coherence using the Sense of Coherence Scale (SOC-13). Among the 1029 residents, a total of 138 (13%) had already returned to Tomioka (group 1), 223 (22%) were undecided (group 2), and 668 (65%) had decided not to return (group 3). Group 2 had poorer HR-QOL than groups 1 and 3, especially physical function, body pain, general health, social functioning, and mental health. The ratio of residents with a better sense of coherence was significantly higher in group 1 than in groups 2 and 3. Our present study indicated poorer HR-QOL among residents who were undecided about returning home. It is necessary to provide a model for a multidisciplinary approach for the public during the recovery phase of a nuclear accident.

## 1. Introduction

Nine years have passed since the Great East Japan Earthquake and the subsequent Fukushima Daiichi Nuclear Power Plant (FNPP) accident on 11 March 2011. It was the largest civilian nuclear accident since the Chernobyl Nuclear Power Plant (CNPP) accident in 1986 [1]. Radionuclides from the damaged plant were released into the environment, and the evacuation order was immediately issued by the Japanese central government to decrease radiation exposure of local residents. The evacuation radius was expanded to within 20 km of the plant the following afternoon [2]. Consequently, 160,000 residents were evacuated. As of 2015, about 67,000 evacuees had moved elsewhere within Fukushima Prefecture and lived in temporary homes, municipally subsidized rentals, and new homes, and about 46,000 evacuees had moved to other prefectures in Japan [3,4,5].

Tomioka Town (37°20′43.6″ N, 141°0′31″ E), which is located within 15 km of the FNPP, was severely damaged by the earthquake and tsunami and was contaminated by radionuclides such as iodine-131, cesium-134, and cesium-137 [4,5]. Immediately after the accident, almost all residents of Tomioka Town were forced to evacuate, mainly to Iwaki City and Koriyama City, 40–60 km away from the town [4,5]. After the termination of the accident, the Tomioka Town municipal government led infrastructure recovery efforts and decontamination processes, and on 1 April 2017, the Japanese government lifted the evacuation order for Tomioka, except for the difficult-to-return zone that comprised almost 15% of the total area of the town [3,4,5]. Although decontamination efforts have decreased radiation levels [5], only about 1400 (11%) of former residents had returned to their homes in Tomioka as of 1 May 2020, 3 years after the evacuation order was lifted. Many residents are reluctant to return to Tomioka. The possible causes of their reluctance almost certainly include concerns over the town’s educational and healthcare infrastructure, as well as convenience in their daily lives. In addition, they might also have been discouraged by risks associated with the decommissioning work being carried out at the FNPP and concerns over the health effects caused by exposure to radiation [6].

Consequently, evacuees have various thoughts about their future. Some of them are set on returning to their homes, while others are eager to re-build their lives in the areas where they are currently living, and others have not yet made a decision [5,7]. It is important to respect the decisions made by individual evacuees regardless of whether they return to their homes or not [8]. It is important for experts to plan and implement measures to support residents that consider the various difficulties that residents have been facing, so that they can be satisfied with their health-related quality of life (HR-QOL) and well-being regardless of whether they return to their homes or not. For healthcare, measuring aspects of HR-QOL such as general health, vitality, and social functioning allows us to think about individual residents as integrated, emotional, and active beings, rather than just organisms. HR-QOL refers to the value of the integrity of life that emphasizes a person’s biographical characteristics and characteristics and is increasingly considered as well-being [9]. After the FNPP accident, Kashiwazaki et al. investigated the relationship between mindfulness and radiation risk perception and the role of health anxiety in Fukushima and Tokyo, and they suggested that mindfulness courses as a part of community support could contribute to the improvement of general health as well as the reduction of health anxiety [10]. In order to respond to the public’s anxiety and health problems, comprehensive community support projects such as managing radiation protection and general health consultations are important regardless of whether residents return to their homes or not. However, the association between HR-QOL and intention to return home has not been estimated. Therefore, in this study, we evaluated the association between HR-QOL and intent to return home among former residents of Tomioka to better understand the current HR-QOL status between returnees and evacuees.

## 2. Materials and Methods

### 2.1. Participants

The study was conducted in Tomioka Town, Fukushima Prefecture in January 2020. The subjects of this study were former residents of Tomioka who had resident cards as of 11 March 2011 and still had them in November 2019, and who were permitted to return to Tomioka Town after the evacuation order was lifted. The residents were not involved in the design, conduct, reporting, or dissemination plans of our research. Minors (aged <20 years old) were not included in this study. We included 1029 former residents of Tomioka (554 men and 475 women) in the analysis. We confirmed that written consent was obtained from the subjects. The study was approved by the ethics committee of Nagasaki University Graduate School of Biomedical Sciences (No. 19092702). Prior to the study, we obtained permission from the municipal government of Tomioka to implement the study.

### 2.2. Questionnaire

The questionnaire applied in this study was developed based on a questionnaire used in previous studies conducted in Fukushima Prefecture [4,5,11] and on the mental health and lifestyle survey within the framework of the Fukushima Health Management Survey, which was organized by Fukushima Prefecture [12,13]. The questionnaire aimed to determine the intent of residents to return home within 5 years of the evacuation order being rescinded. We defined group 1 as “those who had returned to their homes”, group 2 as “those who were undecided about returning home”, and group 3 as “those who had decided not to return home”. We collected data on the demographic variables of sex, age, and living with children aged <18 years. The questionnaire included questions about whether the residents were concerned about consuming locally sourced foods in Tomioka. Furthermore, we assessed perception of the potential health risks of radiation by living in Tomioka, such as cancer and genetic effects, on the next generation.

Quality of life status was assessed using the HR-QOL Short Form-8 (SF-8) scale, which is widely used around the world. The SF-8 is an eight-item instrument that measures general aspects of HR-QOL [14]. The original instrument was developed in the English language and subsequently translated into Japanese. Each administration of the SF-8 generates a health profile with eight dimensions: general health, physical function, role physical (role limitations because of physical health), bodily pain, vitality, social functioning, mental health, and role emotional (role limitations because of emotional problems). The SF-8 incorporates two dimensions: the Physical Component Summary and the Mental Component Summary. Physical Component Summary is composed of four subscales assessing physical function, role limitations caused by physical problems, bodily pain, and general health. Mental Component Summary is composed of four subscales assessing vitality, social functioning, mental health, and role limitations caused by emotional problems. Using the SF-8 to assess HR-QOL has become popular in part because of its ease of administration. The mean value of the Japanese general public was 50 ± 10, which was used to dichotomize the HR-QOL scores in this study [14]. A higher SF-8 score indicates better HR-QOL. We also assessed stress coping among the subjects using the SOC-13, which is widely used in epidemiological and psychological investigations [15]. Sense of coherence is a psychosocial factor that can help individuals be better prepared to maintain and improve their health condition by influencing self-perception and quality of life. The SOC-13 consists of 13 items with answers presented on a seven-point Likert scale. The SOC-13 score is the sum of all the items and ranges from 13 to 91. The higher the score, the stronger the SOC and stress coping. In this study, the criterion “>59 based on the Japanese standard value was used to dichotomize the SOC [15,16].

### 2.3. Statistical Methods

Factors associated with the intention to return home were identified using Mann-Whitney U tests or chi-square tests. Factors that independently differed among groups were identified using logistic regression analysis. Data were statistically analyzed using SPSS (Statistical Package for Social Science) Statistics 25 software (IBM Armonk, NY, USA). A *p*-value less than 0.05 was considered statistically significant.

## 3. Results

Of the 1029 residents investigated, a total of 138 (13%) had returned to Tomioka (group 1), 223 (22%) were undecided (group 2), and 668 (65%) had decided not to return (group 3) (Table 1). The ratio of residents living with children was significantly lower in group 1 than in groups 2 and 3. Compared with group 1, groups 2 and 3 had significantly higher concerns about consuming locally produced food (30.4% vs. 56.1% and 58.8%, respectively). The ratio of residents who felt that cancer would develop in Tomioka due to radiation exposure was significantly higher in groups 2 and 3 than in group 1. Likewise, the ratio of residents who felt that genetic effects would arise in the next generation by returning to Tomioka was significantly higher in groups 2 and 3 than in group 1.

As shown in Table 2, group 2 had poorer HR-QOL than groups 1 and 3, especially physical function, body pain, general health, social functioning, and mental health. The ratio of residents who had better sense of coherence was significantly higher in group 1 than in groups 2 and 3.

We compared group 1 and group 2 using logistic regression analysis (Table 3) and found that the frequency of a better physical component summary score and age were independently higher in group 1 than in group 2. In addition, the frequency of a better Sense of Coherence Scale (SOC-13) score was independently higher in group 1 than in group 2. Similarly, we compared group 1 and group 3 using logistic regression analysis (Table 4) and found that the frequency of poorer HR-QOL status was not significantly different between the groups; however, the frequency of a better SOC-13 score was independently higher in group 1 than in group 3. Finally, we compared group 2 and group 3 using logistic regression analysis (Table 5) and found that the frequency of poor HR-QOL was independently higher in group 2 than in group 3.

## 4. Discussion

Since the Fukushima Daiichi nuclear accident, a series of radiation health risk management projects and research studies have been implemented in Fukushima [1,17]. The United Nations Scientific Committee on the Effects of Atomic Radiation (UNSCEAR) has reported that the exposure doses of the residents of Fukushima were far below of the thresholds for deterministic effects [1]. In addition, UNSCEAR reported that the incidence of health effects in the exposed population would not be expected to be discernible over the baseline level. On the other hand, the present study showed that the frequency of concerns about consuming locally produced foods in Tomioka was significantly higher among residents who were undecided about returning (group 2) and those who had decided not to return (group 3) compared with those who had already returned to Tomioka (group 1). The frequencies of residents who felt that cancer would occur due to radiation exposure and that genetic effects would arise in the next generation due to living in Tomioka were significantly higher in groups 2 and 3 than in group 1. Many previous studies reported that the risk perception of radiation exposure in adults of Fukushima was closely associated with intention to return home [4,18] and psychological distress [5,19,20,21,22]. Here, we showed that residents who had either decided not to return or who were unsure about returning still have high anxiety about radiation exposure and health effects.

The impacts of a major nuclear power plant accident are not limited to the physical health effects of radiation [23]. After the CNPP accident, substantial health and psychological disorders were found among residents [23]. The Chernobyl Forum Report from the 20th anniversary of the CNPP accident concluded that mental health effects were the most significant public health consequence of the accident [24]. First responders and clean-up workers had the greatest exposure to radiation. Recent studies have shown that their rates of depression and post-traumatic stress disorder remain [24,25,26]. General population studies report increased rates of poor self-rated health as well as clinical and subclinical depression, anxiety, and post-traumatic stress disorder (PTSD) [26]. Adults living in contaminated areas around the CNPP had higher incidences of PTSD and other mood and anxiety disorders, as well as significantly lower subjective ratings of health [27]. Thus, long-term mental health consequences continue to be a concern. UNSCEAR also mentioned that mental health problems and impaired social well-being were the major health impacts following the Fukushima Daiichi nuclear accident [1]. In the present study, we showed that group 2 had poorer HR-QOL than groups 1 and 3. Logistic regression analysis revealed the frequency of poorer HR-QOL status was not significantly different between groups 1 and 3, whereas the frequency of poor physical component summary was independently higher in group 2 than in group 1. Similarly, the frequencies of poor physical and mental component summaries were significantly higher in group 2 than in group 3. The possible causes of these results include the fact that residents and evacuees have been facing various difficulties, such as separation from family members, devastation of their home town, and collapse of their community. Evacuees were forced to change various aspects of their lifestyles, such as diet, physical exercise, and other personal habits. [6,27] Residents who were undecided about returning home may be causing poorer HR-QOL due to the difficulty in rebuilding their lives. Specifically, family members could have different opinions, for example, on the physical risk induced by radioactive exposure. Maeda et al. suggested that evacuation or relocation might cause discordance among family members, and these discrepancies in risk perception might be larger among people who live larger distances from Fukushima as evacuees. In addition, they suggested that another perspective of the discordance of family members might come from a sociocultural context. [28]. Our previous study in 2019 evaluated rates of PTSD using a checklist and rates of psychological stress using the patient health questionnaire-9 among residents of Tomioka [5], reporting that residents who were undecided about returning had higher rates of psychological stress and PTSD than those who had returned to their homes and those who had decided not to return. Likewise, in Fukushima, a survey on mental health and lifestyle undertaken among residents of evacuation zones showed a substantial effect of the Fukushima Daiichi nuclear accident on mental health [29]. The survey identified the difficulties of evacuee families, who were separated from each other and moved to unfamiliar areas after the accident, similar to those reported by Chernobyl evacuees. Our current results indicated that former residents who were undecided about returning had not only mental health problems, but also subjective deterioration in well-being. It is important to develop effective intervention methods for residents with poor HR-QOL to help improve their overall well-being.

Many efforts including risk communication during the recovery process were conducted to improve the mental health status of the residents of Fukushima [30,31,32]. Hori et al. performed prolonged exposure therapy for patients with late-onset PTSD affected by evacuation after the FNPP accident [30] and suggested that PTSD symptoms can recur even several years after a disaster due to deterioration of the living environment of a patient. They also reported that an important factor to consider is that the patient should feel safe in his/her daily life. In addition, we [33] have continued risk communication activities and health consultation services regarding radiation and health effects based on the evaluation of internal and external exposure doses of residents in Kawauchi Village, Fukushima Prefecture [33]. Risk communication at the time of a nuclear accident is challenging and needs to address psychological, sociological, and cultural factors that combine to generate public misperceptions about risks [34]. Careful risk communication and dialogue in cooperation with local stakeholders provide support for residents of Fukushima to help improve their mental health status.

In addition, we showed that the frequency of better stress coping was significantly higher in group 1 than in both groups 2 and 3. Murakami et al. evaluated psychological distress among evacuees using the Kessler 6-item Scale in the Fukushima Health Management Survey and reported that returnees had less serious psychological distress than evacuees, even after adjusting for sociodemographic factors [7]. They suggested that living in their original homes might improve the psychological status of affected people. Our results concurred with these findings and also revealed that returnees tend to have better stress coping. Improving stress coping abilities can revive the lives of residents and may improve their well-being. Based on these results, in order to promote the return home of residents who were undecided about returning, we believe that it is important to continue to disseminate information on radiation exposure and to enhance support for the living environment based on individual needs, so that they can feel safe in their daily life after returning to their original home.

Our study had several limitations. First, it was implemented in only one town affected by the Fukushima Daiichi nuclear accident, which might have led to sampling bias. The location of the areas where the participants were living might also cause bias, since Tomioka Town still includes a difficult-to-return zone. Second, since this was a cross-sectional study, any causal relationships between the intention to return home and HR-QOL could not be inferred. There might be other potential factors governing both returning home and HR-QOL. Further investigations are required to determine additional factors contributing to well-being and intention to return among residents, including lifestyle and risk perception. 

## 5. Conclusions

Our present study indicated poorer HR-QOL among residents who were undecided about returning home after the Fukushima Daiichi nuclear accident. In order to promote the return home of residents who were undecided about returning, we believe that it is important to continue to disseminate information on radiation exposure and to enhance support for the living environment based on individual needs, so that they can feel safe in their daily life. It is necessary to provide a model for a multidisciplinary approach for the public during the recovery phase of the Fukushima Daiichi nuclear accident.

## Figures and Tables

**Table 1:** Demographics of groups 1, 2, and 3, and perception of the effects of radiation exposure on heath.

Variables	Reference	Group 1 (*n* = 138)	Group 2 (*n* = 223)	Group 3 (*n* = 668)	*p*-Value
Sex	Male/Female	82/56 (59.4%)	122/101 (54.7%)	350/318 (52.4%)	0.308
Age	≥60/<60 years	105/33 (76.1%)	148/75 (66.4%)	460/208 (68.9%)	0.139
Living with children aged <18 years	Yes/No	9/129 (6.5%)	39/184 (17.5%)	147/521 (22.0%)	<0.001 *
Concerns about consuming locally sourced food	Yes/No	42/96 (30.4%)	125/98 (56.1%)	393/275 (58.8%)	<0.001 *
Belief that living in Tomioka will cause cancer	Yes/No	35/103 (25.4%)	103/120 (46.2%)	362/306 (54.2%)	<0.001 *
Belief that genetic effects will appear in next generation	Yes/No	57/81 (41.3%)	143/80 (64.1%)	413/255 (61.8%)	<0.001 *

Values for groups 1, 2, and 3 are shown as *n* (%). * Significant difference using the chi-squared test.

**Table 2:** HR-QOL (SF-8) and sense of coherence (SOC-13) in groups 1, 2 and 3.

Variables	Domains	Group 1 (*n* = 138)	Group 2 (*n* = 223)	Group 3 (*n* = 668)	*p*-Value
SF-8	Physical functioning	47.7 ± 6.9	46.4 ± 7.2	47.8 ± 7.2	0.043 *
	Role physical	46.6 ± 8.1	45.4 ± 8.5	46.9 ± 8.7	0.085
	Bodily pain	46.9 ± 8.6	44.8 ± 8.8	46.4 ± 9.1	0.046 *
	General health	49.3 ± 7.2	46.3 ± 7.3	48.5 ± 7.5	<0.001 *
	Vitality	49.2 ± 5.9	48.2 ± 6.4	49.1 ± 6.5	0.173
	Social functioning	47.0 ± 8.3	45.2 ± 8.0	46.6 ± 8.0	0.049 *
	Role emotional	47.5 ± 6.6	45.8 ± 7.7	47.0 ± 7.7	0.069
	Mental health	48.3 ± 7.1	45.9 ± 7.0	47.6 ± 7.1	0.003 *
Summary of SF-8	Physical component summary	46.2 ± 7.7	44.8 ± 7.9	46.4 ± 8.1	0.044 *
	Mental component summary	47.8 ± 7.0	45.9 ± 6.8	47.1 ± 7.2	0.034 *
SOC-13	-	60.3 ± 12.2	56.4 ± 12.3	58.1 ± 13.4	0.022 *

* Significant difference using the analysis of variance. HR-QOL (SF-8): Health-Related Quality of Life Short Form 8; SOC-13: Sense of Coherence Scale.

**Table 3:** Factors related to intention to return home according to logistic regression analysis of groups 1 and 2.

Variables	Reference	Model 1		Model 2	
		OR	95% CI	OR	95% CI
Sex	Male/Female	1.12	0.72–1.75	1.08	0.69–1.68
Age	≥60/<60years	1.78 *	1.08–2.94	1.55	0.95–2.52
Physical component summary	Better/Poorer	1.85 *	1.15–2.98	-	-
Mental component summary	Better/Poorer	1.42	0.88–2.24	-	-
SOC-13	Better/Poorer	-	-	1.82 *	1.18–2.82

CI: confidence interval; OR odds ratio; SOC-13: Sense of Coherence Scale. Model 1 analyzes the relationship between intention of return and SF-8 adjusted by sex and age. Model 2 analyzes the relationship between intention of return and SOC-13 adjusted by sex and age. * *p* < 0.05 based on logistic regression analyses.

**Table 4:** Factors related to intention to return home according to logistic regression analysis of groups 1 and 3.

Variables	Reference	Model 1		Model 2	
		OR	95% CI	OR	95% CI
Sex	Male/Female	1.30	0.90–1.89	1.27	0.88–1.86
Age	≥60/<60 years	1.44	0.93–2.23	1.38	0.90–2.11
Physical component summary	Better/Poorer	1.11	0.75–1.64	-	-
Mental component summary	Better/Poorer	0.97	0.66–1.44	-	-
SOC-13	Better/Poorer	-	-	1.62 *	1.12–2.34

CI: confidence interval; OR: odds ratio; SOC-13: Sense of Coherence Scale. Model 1 analyzes the relationship between intention of return and SF-8 adjusted by sex and age. Model 2 analyzes the relationship between intention of return and SOC-13 adjusted by sex and age. * *p* < 0.05 based logistic regression analyses.

**Table 5:** Factors related to intention to return home according to logistic regression analysis of groups 2 and 3.

Variables	Reference	Model 1		Model 2	
		OR	95% CI	OR	95% CI
Sex	Male/Female	1.14	0.83–1.55	1.12	0.82–1.52
Age	<60/≥60 years	1.20	0.86–1.68	1.12	0.81–1.55
Physical component summary	Poorer/Better	1.63 *	1.55–2.30	-	-
Mental component summary	Poorer/Better	1.42 *	1.01–1.99	-	-
SOC-13	Poorer/Better	-	-	1.13	0.82–1.55

CI: confidence interval; OR: odds ratio; SOC-13: Sense of Coherence Scale. Model 1 analyzes the relationship between intention of return and SF-8 adjusted by sex and age. Model 2 analyzes the relationship between intention of return and SOC-13 adjusted by sex and age. * *p* < 0.05 based on logistic regression analyses.
